# Where do you live? North versus Central-South differences in relation to Italian patients with oral lichen planus: a cross-sectional study from the SIPMO (Italian Society of Oral Pathology and Medicine)

**DOI:** 10.1186/s12903-022-02181-7

**Published:** 2022-05-18

**Authors:** Daniela Adamo, Elena Calabria, Federica Canfora, Noemi Coppola, Lorenzo Lo Muzio, Francesca Spirito, Michele Giuliani, Lorenzo Azzi, Vittorio Maurino, Giuseppe Colella, Chiara Colella, Lucio Montebugnoli, Davide Bartolomeo Gissi, Mario Gabriele, Marco Nisi, Andrea Sardella, Giovanni Lodi, Elena Maria Varoni, Amerigo Giudice, Alessandro Antonelli, Paolo Giacomo Arduino, Alessio Gambino, Paolo Vescovi, Alessandra Majorana, Elena Bardellini, Giuseppina Campisi, Vera Panzarella, Francesco Spadari, Umberto Garagiola, Monica Pentenero, Samuele Sutera, Matteo Biasotto, Giulia Ottaviani, Margherita Gobbo, Luca Guarda Nardini, Umberto Romeo, Gianluca Tenore, Rosario Serpico, Alberta Lucchese, Carlo Lajolo, Cosimo Rupe, Massimo Aria, Luca D’Aniello, Michele Davide Mignogna, SIPMO (Italian Society of Oral Pathology and Medicine)

**Affiliations:** 1 Department of Neuroscience, Reproductive Sciences and Dentistry, University of Naples Federico II, Naples, Italy; 2 Department of Clinical and Experimental Medicine, University of Foggia, Foggia, Italy; 3 Unit of Oral Medicine and Pathology, ASST dei Sette Laghi, Department of Medicine and Surgery, University of Insubria, Varese, Italy; 4 Multidisciplinary Department of Medical, Surgical and Dental Specialties, University of Campania Luigi Vanvitelli, Naples, Italy; 5 Department of Biomedical and Neuromotor Sciences, Section of Oral Sciences, University of Bologna, Bologna, Italy; 6 Department of Surgical Pathology, Medicine, Molecular and Critical Area, University of Pisa, Pisa, Italy; 7 Department of Biomedical, Surgical and Dental Sciences, University of Milan, Milan, Italy; 8 Department of Health Sciences, Magna Graecia University of Catanzaro, Catanzaro, Italy; 9 Oral Medicine Section, Department of Surgical Science, CIR Dental School, University of Turin, Turin, Italy; 10 Department of Medicine and Surgery, Oral Medicine and Laser Surgery Unit, University Center of Dentistry, University of Parma, Parma, Italy; 11 Department of Medical and Surgical Specialities, Radiological Sciences and Public Health, University of Brescia, Brescia, Italy; 12 Department of Surgical, Oncological, and Oral Sciences, University of Palermo, Palermo, Italy; 13 Department of Biomedical, Surgical and Dental Sciences, Maxillo-Facial and Dental Unit, Fondazione IRCCS Ca’ Granda Ospedale Maggiore Policlinico, University of Milan, Milan, Italy; 14 Department of Oncology, Oral Medicine and Oral Oncology Unit, University of Turin, Turin, Italy; 15 Department of Medical, Surgical and Health Sciences, University of Trieste, Trieste, Italy; 16 Unit of Oral and Maxillofacial Surgery, Ca’ Foncello Hospital, Treviso, Italy; 17 Department of Oral Sciences and Maxillofacial Surgery, University of Rome La Sapienza, Rome, Italy; 18 Head and Neck Department, Fondazione Policlinico Universitario A. Gemelli IRCCS, School of Dentistry, Università Cattolica del Sacro Cuore, Rome, Italy; 19 Department of Economics and Statistics, University Federico II of Naples, Naples, Italy; 20 Department of Social Sciences, University Federico II of Naples, Naples, Italy

**Keywords:** Oral lichen planus, Psychological profile, Sleep disturbances, Pain, Italy

## Abstract

**Background:**

Oral lichen planus (OLP) is an immune-mediated inflammatory chronic disease of the oral mucosa, with different patterns of clinical manifestations which range from keratotic manifestations (K-OLP) to predominantly non-keratotic lesions (nK-OLP). The aim of the study was to analyze the differences in the clinical, psychological profile and symptoms between Italian patients of the North and Central-South with K-OLP and nK-OLP.

**Methods:**

270 K-OLP and 270 nK-OLP patients were recruited in 15 Italian universities. The Numeric Rating Scale (NRS), Total Pain Rating Index (T-PRI), Hamilton Rating Scales for Depression and for Anxiety (HAM-D and HAM-A), Pittsburgh Sleep Quality Index (PSQI), and Epworth Sleepiness Scale (ESS) were administered.

**Results:**

The Central-South K-OLP (CS-K-OLP) patients reported a higher frequency of pain/burning compared with the K-OLP patients of the North (N-K-OLP) with higher scores in the NRS and T-PRI (*p* value < 0.001**). The CS-K-OLP and the CS-nK-OLP patients showed higher scores in the HAM-D, HAM-A, PSQI and ESS compared with the Northern patients (*p* value < 0.001**). Multivariate logistic regression revealed that the NRS and T-PRI showed the greatest increase in the R2 value for the CS-K-OLP (DR2 = 9.6%; *p* value < 0.001**; DR2 = 9.7% *p* value < 0.001**; respectively) and that the oral symptoms (globus, itching and intraoral foreign body sensation) and PSQI showed the greatest increase in the R2 value for the CS-nK-OLP (DR2 = 5.6%; *p* value < 0.001**; DR2 = 4.5% *p* value < 0.001** respectively).

**Conclusions:**

Pain and mood disorders are predominant in patients with OLP in the Central-South of Italy. Clinicians should consider that the geographical living area may explain the differences in oral symptoms and psychological profile in OLP.

## Background

Oral lichen planus (OLP) is a T-cell–mediated inflammatory chronic disorder affecting the oral mucosa [1]. The cause of the disease remains unknown but genetic dysfunctions, environmental factors and mood disorders such as stress, anxiety, depression and sleep disturbances have a role in activating the immunological system [1]. The global OLP prevalence, 1.01%, reveals geographical differences with the highest prevalence in Europe (1.43%) and a higher incidence in women than in men [2]. The affected age range is variable around the world, with a risk of developing the disease 3.43 times higher in patients ≥ 40 years [2].

OLP may present with different clinical patterns ranging from keratotic manifestations (K-OLP, white reticular, papular and/or plaque-like lesions) to predominantly non-keratotic lesions (nK-OLP, atrophic, erythematous, erosive, ulcerative and/or bullous lesions) [3, 4]. The patients commonly reported a complex symptomatology characterized by pain/burning and additional oral symptoms, such as xerostomia, dysgeusia and globus, with nK-OLP patients reporting a higher level of pain compared with K-OLP patients [4]. A greater prevalence of anxiety, depression and stress has been reported in OLP patients compared with the general population, which may contribute not only to the onset but also to the exacerbation of the disease, impairing in turn the patient’s quality of life [5].

A sociodemographic analysis and a description of the clinical characteristics of OLP have been provided in some studies, predominantly with a retrospective design [6–8]. However, none of these studies have included a psychological profile evaluation in relation to these patients. In Italy, two studies have been performed across the years. Carbone et al. [9] evaluated a wide cohort of patient with OLP living in northern Italy while Lauritano et al. [10] evaluated a cohort of eighty-seven southern Italian patients with OLP. However, to the best of our knowledge, no studies have evaluated the differences in socio-demographic parameters, psychological profile and symptomatology in patients affected by OLP in distinct geographical areas of a country. Until now, medical research has been focused on the comorbidities and illness duration of the patients with little consideration of the implications that different geographical area of hospitalization could have with respect to a medical condition. Indeed, the “living place”, through the interaction between genetic factors, lifestyle and environment, may affect various aspects of a disease.

Therefore, the aim of the study has been to analyze the socio-demographic and clinical characteristics, psychological profile and symptoms in a large cohort of 540 Italian patients with OLP, in order to evaluate any similarities and differences between K-OLP and nK-OLP patients living in the geographical areas of the North (N) and Central-South (CS) of Italy.

## Materials and methods

### Study design

This was a descriptive secondary analysis of an observational multicenter study carried out between December 2018 and January 2020 in fifteen Oral Medicine outpatients' departments of different Italian Universities with the participation of the Italian Society of Oral Pathology and Medicine (SIPMO- Società Italiana di Patologia e Medicina Orale). The study was conducted in accordance with the ethical principles of the World Medical Association Declaration of Helsinki and was approved by the Ethics Committee of Federico II University of Naples, the chief investigator center (reference number: 184/18). All the other Oral Medicine departments which participated in the study had to obtain the ethical approval of their local ethics committees. The reporting of data followed the Strengthening the Reporting of Observational Studies in Epidemiology (STROBE) guidelines for observational studies [11].

### Participants

Overall, the study group consisted of 270 K-OLP and 270 nK-OLP patients, equally distributed between the North and Central-South. All the enrolled patients provided their written informed consent to participate in the study. The patients were recruited considering the following inclusion and exclusion criteria.

The inclusion criteria were:

1. Patients of either gender, aged 18 or older
2. Patients with a clinical and histopathological diagnosis of OLP based upon the modified WHO diagnostic criteria [12]
3. Patients with an exclusive keratotic pattern (reticular, papular and/or plaque-like lesions) or a prevalent non-keratotic pattern (a predominant atrophic, erythematous, erosive, ulcerative, and /or bullous phenotype). The former patients were included in the K-OLP group and the latter patients in the nK-OLP group.

The exclusion criteria were:

1. Pregnant or breastfeeding women
2. Evidence of oral epithelial dysplasia
3. A suspicion that the oral lesions may be related to drug-taking or a contact with dental materials
4. The presence of oral mucosal disease
5. The presence of a coexisting autoimmune disease, tumor or a history or occurrence of psychiatric illness as defined by the American Psychiatric Association Diagnostic and Statistical Manual of Mental Disorders 5 (DSM-5)
6. A history of alcohol or substance abuse
7. Ongoing treatment with systemic and/or topical corticosteroids or with psychotropic drugs
8. An inability to understand the questionnaires

The patients first received a complete clinical interview for the collection of data relating to their sociodemographic status and health-related factors, such as comorbidities and drug intake, using a structured questionnaire. Each patient underwent a complete intra- and extra-oral examination, as previously described [4]. On the basis of clinical findings of OLP, the patients were categorized into one of the two groups described above (K-OLP and nK-OLP). The site of the oral mucosa involved by OLP and the site and type of the oral symptoms were recorded.

The following set of validated questionnaires, in their Italian versions, were administered to all the patients:

- the Numeric Rating Scale (NRS) and Total Pain Rating Index (T-PRI) from the Short Form of the McGill Pain Questionnaire (SF-MPQ) for the assessment of the oral discomfort, and the intensity and quality of pain [13, 14];
- the Hamilton Rating Scale for Depression (HAM-D) and the Hamilton Rating Scale for Anxiety (HAM-A) for the evaluation of depression and anxiety [15, 16];
- the Pittsburgh Sleep Quality Index (PSQI) and the Epworth Sleepiness Scale (ESS) for the assessment of sleep [17, 18].

### Statistical analyses

In our previous studies [4, 19], the sample consisted of a total of 600 OLP patients, 330 from the northern regions and 270 from the central-south areas. Therefore, in order to include the same number of patients for each subgroup (Central-South-OLP vs North OLP patients), the patients from the two groups were matched based on gender and age. Ultimately, a total of 60 patients were excluded from the northern group.

The sample size was set by fixing a test power of no less than 90% associated with a significance of no more than 5% (software G*Power 3.1.9.7 by Dusseldorf University). This sample size calculation was performed using the effect size estimation from a previously published research study regarding the same questionnaires for pain, sleep and mood disorders. To remove the confounding effects of significant socio-demographic characteristics, a statistical matching approach [20], based on the nearest neighbor distance, was applied in advance to compare the sleep quality, anxiety and depression scores between the patients and controls [20].

The statistical analyses were performed based on our previous research [4, 19] by using the SPSS software v. 23. Descriptive statistics, including means, standard deviations, medians and the interquartile range (IQR), were used to analyze the socio-demographic and clinical characteristics of the four subgroups. The Pearson Chi Square test, Fisher’s exact test were used to test the significance differences between the percentages in the four subgroups. Differences associated with p values less than 0.05 or 0.01 were considered moderately or strongly significant, respectively. The non‐parametric ANOVA procedure by Mann–Whitney test was employed to test for any differences between the recorded medians of the HAM‐D, HAM‐A, PSQI, ESS, NRS and T-PRI in the groups. *P* values < 0.05 were considered to reflect a statistical significance. In order to estimate the presence of potential predictors of the Central-South geographical area provenance, multiple linear regression analyses were performed, in both the K-OLPs and nK-OLPs, considering sociodemographic variables, smoking, alcohol use, BMI, oral symptoms, oral sites of OLP lesions, pain intensity and quality (NRS and TPR-I), the psychological profile (HAM-A and HAM-D) and the quality of sleep (PSQI and ESS). Full models, when all the variables were entered simultaneously, were used to evaluate the relative contributions of these variables.

In detail, a sequential regression model analysis including the predictors, one by one, to obtain unadjusted coefficient estimations was performed. Moreover, as a final step, a full model analysis considering all the predictors, simultaneously, to estimate the adjusted coefficients was carried out. In all the steps, we provided standard errors of model coefficients, which measure the statistical precision of an inference estimation of the model parameters.

## Results

A total of 540 participants were included, 270 OLP patients from the North Italian area and 270 OLP patients from the Central-South. Both groups were equally composed of 135 K-OLP and 135 nK-OLP patients.

A comparison of the sociodemographic characteristics of the patients is shown in Table 1. 179 patients with OLP (65%) were female. No statistically significant differences were found between the North and Central-South K-OLP and nK-OLP patients in relation to gender, employment, family status, age, BMI, smoking and alcohol consumption. However, the CS-nK-OLP patients presented a higher level of education compared to the N-nK-OLPs (*p* value: 0.048*), while no difference was detected among the K-OLP patients. On the other hand, a statistically significant higher proportion of the N-K-OLP patients were found to be habitual alcohol consumers compared to the CS-K-OLP patients (*p* value: 0.001**).

**Table 1:** North versus Central-South differences between 270 K-OLP and 270 nK-OLP in sociodemographic profile, BMI, and risk factors

Demographic variables	K-OLPs (N:270)		*p* value	nK-OLPs (N:270)		*p* value
	Nord (N:135)	Central/South (N:135)		Nord (N:135)	Central/South (N:135)	
	N/Frequency (%)			N/Frequency (%)		
**Gender**						
Male	57 (42.2)	48 (35.6)	0.318	37 (27.4)	46 (34.1)	0.291
Female	78 (57.8)	87 (64.4)		98 (72.6)	89 (65.9)	
**Employment**						
Employed	55 (40.7)	50 (37)	0.054	38 (28.1)	40 (29.6)	0.375
Unemployed	27 (20)	44 (32.6)		23 (17)	31 (23)	
Retired	53 (39.3)	41 (30.4)		74 (54.8)	64 (47.4)	
**Family situation**						
Single	17 (12.6)	13 (9.6)	0.102	12 (8.9)	10 (7.4)	0.686
Married	104 (77)	98 (72.6)		103 (76.3)	98 (72.6)	
Divorced	3 (2.2)	12 (8.9)		7 (5.2)	8 (5.9)	
Widowed	11 (8.1)	12 (8.9)		13 (9.6)	19 (14.1)	

	Mean ± SD			Mean ± SD		
**Age** (in years)	64 ± 10.2	63.5 ± 12.1	0.835	64.9 ± 9.77	64 ± 11.1	0.407
**Education** (in years)	11 ± 4.01	11.2 ± 3.79	0.594	10.7 ± 4.38	11.7 ± 4.04	0.048*
**Body mass index**	25 ± 4.22	25 ± 3.77	0.552	24.9 ± 3.9	25.4 ± 4.09	0.249

Risk factors	N/Frequency (%)			N/Frequency (%)		
**Smoking**						
Yes	30 (22.2)	35 (25.9)	0.569	22 (16.3)	34(25.2)	0.099
No	105 (77.8)	100 (74.1)		113 (83.79	101(74.8)	
**Alcohol consumption**						
Yes (≤ 14 units/week)	64 (47.4)	37 (27.4)	0.001**	57 (42.2)	42(31.1)	0.077
No	71 (52.6)	98 (72.6)		78 (57.8)	93(68.9)	

The significance difference among the percentages was measured by the Pearson Chi Square test. *Significant 0.01 < *p* ≤ 0.05, **Significant *p* ≤ 0.01

The significance difference among the percentages of family situation was measured by the Fisher’s exact test. *Significant 0.01 < *p* ≤ 0.05, **Significant *p* ≤ 0.01

*K-OLP* keratotic oral lichen planus, *nK-OLP* non-keratotic oral lichen planus

The frequencies of systemic comorbidities and the drugs used by the patients are shown in detail in Table 2. No statistically significant differences were found in the frequency of systemic diseases in the OLP patients living in the different geographical areas. In detail, 79 (63.2%) N-K-OLP and 72 (53.7%) CS-K-OLP patients (*p* value: 0.132) and 116 (85.9%) N-nK-OLP and 108 (80.0%) CS-nK-OLP patients (*p* value: 0.257) presented systemic comorbidities. With respect to drug intake, there was a statistically significant difference in terms of the percentage of patients taking systemic medication among the nK-OLP patients (*p* value: 0.005**), with a higher prevalence in the N-nK-OLP patients (105; 77.8%) compared with the CS-nK-OLP patients (83; 61.5%). No differences were detected in drug consumption among the K-OLP patients [N-K-OLP: 88(65.2%); CS-K-OLP: 93(65%); *p* value: 0.605]. A higher proportion of the N-nK-OLP patients suffered from gastro-esophageal reflux disease (*p* value: 0.036*) compared to the CS-nK-OLP patients, who in turn presented statistically significant higher frequencies of diuretic and blood thinner intake (*p* values: 0.008**and 0.005** respectively). On the contrary, no statistically significant difference was found among the K-OLP patients.

**Table 2:** North versus Central-South differences between 270 K-OLP and 270 nK-OLP in systemic diseases and drug consumption

	K-OLPs (N:270)		*p* value	nK-OLPs (N:270)		*p* value
	Nord (N:135)	Central/South (N:135)		Nord (N:135)	Central/South (N:135)	
	N/Frequency (%)			N/Frequency (%)		
**Systemic diseases**						
Essential hypertension	40 (29.6)	50 (37.9)	0.132	61 (45.2)	67 (49.6)	0.542
Hypercholesterolemia	26 (19.3)	32 (23.7)	0.245	28 (20.07)	35 (25.9)	0.388
Previous myocardial infarction	2 (1.5)	2 (1.5)	0.459	3 (2.2)	2 (1.5)	1
Diabetes mellitus type 2	9 (6.6)	14 (10.3)	0.458	11 (8.1)	15 (11.1)	0.825
Asthma	2 (1.5)	3 (2.2)	1	5 (3.7)	9 (6.7)	0.411
Gastro-esophageal reflux disease	17 (12.6)	22 (16.3)	0.489	36 (26.7)	21 (15.6)	0.036*
Hepatitis B	3 (2.2)	0 (0)	0.247	0 (0)	1 (0.7)	1
Hepatitis C	3 (2.2)	3 (2.2)	1	4 (3.0)	3 (2.2)	1
Endocrine disease	4 (3.0)	7 (5.2)	0.54	5 (3.7)	6 (4.4)	0.411
Hypothyroidism	17 (12.6)	17 (12.6)	1	19 (14.1)	11 (8.1)	0.174
Hyperthyroidism	2 (1.5)	3 (2.2)	1	4 (3.0)	3 (2.2)	1
Benign prostatic hypertrophy	7 (5.2)	9 (6.7)	0.798	7 (5.2)	8 (5.9)	1
Previous malignant disease	12 (8.9)	11 (8.1)	1	12 (8.9)	11 (8.1)	1
**Drug consumption**						
Beta‐Adrenergic receptor blockers	17 (12.6)	23 (17.0)	0.392	38 (28.1)	22 (16.3)	0.028
Angiotensin II receptor blockers	11 (8.1)	11 (8.1)	1	12 (8.9)	9 (6.7)	0.65
Diuretics	7 (5.2)	13 (9.6)	0.245	5 (3.7)	18 (13.3)	0.008**
Calcium channel blockers	6 (4.4)	8 (5.9)	0.785	14 (10.4)	10 (7.5)	0.522
ACE-inhibitors	10 (7.4)	16 (11.9)	0.302	24 (17.8)	29 (21.5)	0.54
Simvastatin	14 (10.4)	21 (15.6)	0.277	23 (17)	30 (22.2)	0.358
Metformin	7 (5.2)	15 (11.1)	0.118	7 (5.2)	11 (8.1)	0.465
Insulin	3 (2.2)	5 (3.7)	0.772	3 (2.2)	4 (3.0)	1
Antiplatelets	17 (12.6)	9 (6.7)	0.148	17 (12.6)	20 (14.8)	0.724
Blood thinners	4 (3.0)	10 (7.4)	0.168	1 (0.7)	11 (8.1)	0.005**
Levothyroxine sodium	16 (11.9)	20 (14.8)	0.592	19 (14.1)	13 (9.6)	0.347
Proton pump inhibitors	14 (10.4)	20 (14.8)	0.359	26 (19.3)	29 (21.5)	0.763

The significance difference among the percentages was measured by the Fisher’s exact test. *Significant 0.01 < *p* ≤ 0.05, **Significant *p* ≤ 0.01

*K-OLP* keratotic oral lichen planus, *nK-OLP* non-keratotic oral lichen planus

Regarding the clinical presentation, diffuse OLP lesions which affected all the oral mucosal sites (gingiva, lips, buccal mucosa, tongue, floor of the mouth, palate and retromolar trigone) were detected in 27 (20%) N-K-OLP and 13 (9.6%) CS-K-OLP patients (*p* value 0.025*) and in 10 (7.4%) N-nK-OLP and 12 (8.9%) CS-nK-OLP patients (*p* value: 0.825), while in the majority of the cases only some sites were involved. The CS-OLP patients presented a higher number of sites involved by OLP lesions compared with the N-OLP patients. However, this difference was found to be statistically significant only between the CS-nK-OLP and N-nK-OLP patients (*p* value: 0.007**). Table 3 shows the details of the frequency distribution of the oral sites and oral symptoms involved by the OLP lesions. The most frequent sites involved by the OLP lesions were the buccal mucosa (244; 42.8%), the gingiva (223; 39.1%) and the tongue (212; 37.1%). A statistically significant difference was observed in the prevalence of lesions of the retromolar trigone between both groups, the K-OLP and nK-OLP. Indeed, a higher prevalence of lesions of the retromolar trigone was found in the N-K-OLP compared with the CS-K-OLP patients (33, 24.4% and 18, 13.3% respectively; *p* value: 0.029*) and in the CS-nK-OLP compared with the N-K-OLP patients (29, 21.5% and 12, 8.9% respectively; *p* value: 0.006**). In addition, a statistically significant difference was found in the prevalence of lesions of the floor of the mouth with a higher prevalence in the CS-nK-OLP patients (28, 20.7%). For most of these patients, the pain was described as burning in character and was the most common reported symptom. The nK-OLP patients (181, 67%) were more symptomatic compared with the K-OLP patients (133, 49%) and the CS-OLP patients were more symptomatic compared with the patients of the North area. Indeed, the pain/burning was reported by 173 (64%) of the CS-OLP and 141 (52%) of the N-OLP patients. A statistically significant difference was found in the pain/ burning perception in the K-OLP patients [N-K-OLP: 48 (35.6%); CS- K-OLP: 85 (63.0%), *p* value: < 0.01**] while, despite the high prevalence of pain/burning, no statistically significant difference was found in the nK-OLP patients [N-nK-OLP: 93(68.9%) and CS-nK-OLP: 88 (65.2%), *p* value: 0.605]. Moreover, the CS-K-OLP patients showed a statistically significant difference in the prevalence of additional oral symptoms reported, such as xerostomia, itching and tingling sensations, in comparison with the N-K-OLP patients (*p* values: 0.037*, 0.037*and 0.022* respectively). In addition, the CS-nK-OLP patients showed a statistically significant difference in terms of globus pharyngeus, itching and intra-oral foreign body sensation compared with the N-nK-OLP patients (*p* values: 0.031*, 0.013* and 0.021* respectively).

**Table 3:** North versus Central-South differences between 270 K-OLP and 270 nK-OLP in oral sites involved, and oral symptoms

	K-OLPs (N:270)		*p* value	nK-OLPs (N:270)		*p* value
	Nord (N:135)	Central/South (N:135)		Nord (N:135)	Central/South (N:135)	
	N/Frequency (%)			N/Frequency (%)		
**Oral sites involved by OLP**						
Gingiva	48 (35.5)	56 (41.5)	0.381	54 (40.0)	65 (48.1)	0.22
Lips	37 (27.4)	40 (29.6)	0.788	24 (17.8)	38 (28.1)	0.059
Buccal mucosa	58 (43.3)	62 (45.9)	0.073	60 (44.4)	64 (47.4)	0.714
Tongue	56 (41.5)	52 (38.5)	0.713	44 (32.6)	60 (44.4)	0.06
Floor of the mouth	35 (25.9)	23 (17.0)	0.709	13 (9.7)	28 (20.7)	0.017*
Palate	46 (34.1)	33 (24.4)	0.108	33 (24.4)	36 (26.7)	0.78
Retromolar trigone	33 (24.4)	18 (13.3)	0.029*	12 (8.9)	29 (21.5)	0.006*

	Median; [IQR]		*p* value	Median; [IQR]		*p* value
Number of sites involved	1; [0–5]	2; [1–3]	0.062	1; [1, 2]	2; [1–3]	0.007**
Oral symptoms	N/Frequency (%)		*p* value	N/Frequency (%)		N/Frequency (%)
Pain/burning	48 (35.6)	85 (63.0)	< 0.01**	93 (68.9)	88 (65.2)	0.605
Xerostomia	38 (28.4)	50 (37.0)	0.037*	38 (28.1)	54 (40)	0.054
Dysgeusia	18 (13.3)	28 (20.7)	0.145	25 (18.5)	30 (22.2)	0.546
Sialorrhea	11 (8.1)	17 (12.6)	0.318	24 (17.8)	25 (18.5)	1
Subjective halitosis	22 (16.3)	25 (18.5)	0.748	27 (20.0)	28 (20.7)	1
Globus pharyngeus	16 (11.9)	18 (13.3)	0.855	15 (11.1)	29 (21.5)	0.031*
Itching	10 (7.5)	22 (16.3)	0.037*	9 (6.7)	23 (17.0)	0.013*
Intraoral foreign body sensation	13 (9.6)	17 (12.6)	0.562	9 (6.7)	22 (16.3)	0.021*
Tingling sensation	7 (5.2)	19 (14.1)	0.022*	14 (10.4)	19 (14.1)	0.458
Occlusal dysesthesia	11 (8.1)	10 (7.4)	1	8 (5.9)	12 (8.9)	0.487
Change in tongue morphology	1 (0.7)	0 (0)	1	2 (1.5)	0 (0)	0.498
Oral dyskinesia	2 (1.5)	2 (1.5)	1	1 (0.7)	6 (4.4)	0.12
Dysosmia	9 (6.7)	5 (3.7)	0.411	6 (4.4)	10 (7.4)	0.44

The significance difference between percentages was measured by Fisher’s exact test. *Significant 0.01 < *p* ≤ 0.05, **Significant *p* ≤ 0.01

*K-OLP* keratotic oral lichen planus, *nK-OLP* non-keratotic oral lichen planus

Comparisons of the clinical parameters between the North and Central-South OLP patients are summarized in Table 4. The OLP patients of the Central-South area presented statistically significant higher levels of anxiety, depression and sleep disturbances compared to the Northern group (*p* value < 0.001**). Interestingly, only the CS-OLP patients with the keratotic phenotype presented statistically significant higher median scores in the NRS and T-PRI in comparison with the N K-OLPs (*p* value: < 0.001**), while this difference was not recorded among the OLP patients suffering from the non-keratotic phenotype (*p* value: 0.175).

**Table 4:** North versus Central-South differences between 270 K-OLP and 270 nK-OLP in pain, depression, anxiety and sleep disturbance

	K-OLPs (N:270)		*p* value	nK-OLPs (N:270)		*p* value
	Nord (N:135)	Central/South (N:135)		Nord (N:135)	Central/South (N:135)	
	Median; [IQR]			Median; [IQR]		
**Total score of test**						
NRS	0.0; [0–2.5]	4.0; [0–6]	< 0.001**	3.0; [0 – 6]	5.0; [0–7]	0.175
T-PRI	1.0; [0.2]	3.0; [0–6]	< 0.001**	3.0; [1–6]	3.0; [0.5–7]	0.427
HAM-D	5.0; [1.5–9]	8.0; [4–14]	< 0.001**	7.0; [4–10]	10 [5–17]	< 0.001**
HAM-A	5.0; [2–9]	9.0; [5–12.5]	< 0.001**	6.0; [3–12.5]	12 [5.5–18]	0.001**
PSQI	4.0; [3–6]	6.0; [4–8]	< 0.001**	5.0; [3–8]	7 [5–10]	< 0.001**
ESS	4.0; [1.5–6]	6.0; [3–9]	< 0.001**	4.0; [2–7]	7; [3–9.5]	0.002**

	N/Frequency (%)			N/Frequency (%)		
**Psychological profile**						
Depression (HAM-D ≥ 7)	44 (32.6.)	75 (55.6)	< 0.001**	60 (44.4)	81 (60.0)	0.015*
Anxiety (HAM-A ≥ 7)	45 (33.7)	84 (62.2)	< 0.001**	59 (43.7)	85 (63.0)	0.002**
Sleep Disturbance (PSQI ≥ 5)	48 (35.6)	79 (58.5)	< 0.001**	55 (40.7%)	85 (63.0)	< 0.001**
Daytime sleepiness (ESS ≥ 10)	15 (11.1)	23 (17.0)	0.221	25 (18.5)	34 (25.2)	0.239

The significance difference among the medians was measured by the Mann–Whitney test. *Significant 0.01 < *p* ≤ 0.05, **Significant *p* ≤ 0.01

The significance difference among the percentages was measured by the Pearson Chi Square test. *Significant 0.01 < *p* ≤ 0.05, **Significant *p* ≤ 0.01

*ESS* Epworth sleepiness scale, K-OLP keratotic oral lichen planus, *nK-OLP* non-keratotic oral lichen planus, *HAM-A* Hamilton anxiety, *HAM-D* Hamilton depression, *PSQI* Pittsburgh sleep quality index, *T-PRI* Total pain rating index

The analysis of the psychological profiles showed a higher prevalence of anxiety (HAM-A ≥ 7) in the OLP patients of the Central-South area compared with the OLP patients of the North [CS-K-OLP: 84(62.2%); N-K-OLP: 45(33.7%), *p* value < 0.001**; CS-nK-OLP: 85(63%); N-nK-OLP: 59(43.7%) *p* value: 0.002**]. In addition, a higher prevalence of poor sleepers (PSQI > 5) was found in the OLP patients of the Central-South the area compared with the OLP patients of the North [N-K-OLP: 48(35.5%); CS-K-OLP: 79 (58.5%), *p* value < 0.001**; N-nK-OLP: 55 (40.7%); CS-nK-OLP: 85(63%) *p* value < 0.001**]. Excessive sleepiness (ESS > 10) was found in 40 (14.8%) N-OLP and in 57 (21%) CS-OLP patients.

The results of the simultaneous multiple linear regression analyses for the K-OLP and nK-OLP groups, predicting the Central-South area, are shown in Tables 5 and 6 respectively. The first model (the sociodemographic model) tested the contributions of the demographic variables and habits, with only alcohol intake being found to be negatively correlated in both the K-OLP and nK-OLP patients (Beta: − 1.12; *p* value: 0.001**, − 1.02, *p* value: 0.001** respectively), resulting in a significant increase in the R2 value (K-OLP: *p* value: 0.015*, nK-OLP: *p* value:0.009**). With respect to the symptoms and oral sites involved, only those resulting statistically different were tested for their contribution (burning, itching, tingling and retromolar trigone were evaluated for the K-OLP patients and globus pharingeus, itching, intraoral body sensation, floor of the mouth and retromolar trigone were evaluated for the nK-OLP patients). The addition of the symptoms in the second model (the symptoms model) resulted in a significant increase in the R2 values in both the K-OLP and nK-OLP patients (K-OLP: DR2 = 6.1%; *p* value < 0.001**; nK-OLP DR2 = 5.6%; *p* value < 0.001**). The addition of the oral sites involved by OLP lesions in the third model (the clinical model) resulted in a significant increase in the R2 values in both K-OLP and nK-OLP patients (K-OLP: DR2 = 1.8%; *p* value 0.044*; nK-OLP: DR2 = 3.3%; *p* value 0.006**). The addition of the NRS in the fourth model (the pain intensity model) resulted in a significant increase in the R2 values in both OLP groups (K-OLP: DR2 = 9.6%; *p* value < 0.001**; nK-OLP: DR2 = 1.9. *p* value 0.034*); the addition of the T-PRI in the fifth model (the pain quality model) resulted in a significant increase in the R2 values only in the K-OLP patients (K-OLP: DR2 = 9.7%; *p* value < 0.001**). The addition of the HAM-D in the sixth model (the depression model) resulted in a significant increase in the R2 values in both the K-OLP and nK-OLP patients (K-OLP DR2 = 3.1%; *p* value < 0.001**; nK-OLP DR2 = 4.3%; *p* value < 0.001**). The addition of the HAM-A in the seventh model (the anxiety model) resulted in a significant increase in the R2 values in both the K-OLP and nK-OLP patients (K-OLP DR2 = 2.4%; *p* value 0.004**; nK-OLP DR2 = 4.1%; *p* value < 0.001**). The addition of the PSQI in the eighth model (the sleep model) resulted in a significant increase in the R2 values in both the K-OLP and nK-OLP patients (K-OLP DR2 = 3.9%; *p* value < 0.001**; nK-OLP DR2 = 4.5%; *p* value < 0.001**). The addition of the ESS in the ninth model (the sleepiness model) resulted in a significant increase in the R2 values in both the K-OLP and nK-OLP patients (K-OLP DR2 = 2.7%; *p* value 0.002**; nK-OLP DR2 = 2.0%; *p* value 0.009**).

**Table 5:** Multivariate Logistic regression analysis predicting geographic area (target area Central-south = 1) in 270 K-OLP patients

Predictors	Model 1			Model 2			Model 3			Model 4			Model 5		
	Beta (SE)	OR	*p* value	Beta (SE)	OR	*p* value	Beta (SE)	OR	*p* value	Beta (SE)	OR	*p* value	Beta (SE)	OR	*p* value
Age	− 0.01 (0.02)	0.99	0.612	− 0.01 (0.02)	0.99	0.659	0.00 (0.02)	1.00	0.781	− 0.01 (0.02)	0.99	0.551	− 0.01 (0.02)	0.99	0.781
Gender: female	0.24 (0.29)	1.27	0.413	0.01 (0.31)	1.01	0.970	0.18 (0.30)	1.20	0.543	0.02 (0.31)	1.02	0.951	0.18 (0.30)	1.20	0.550
Years of education	0.05 (0.04)	1.05	0.225	0.04 (0.04)	1.04	0.362	0.04 (0.04)	1.04	0.282	0.05 (0.04)	1.05	0.207	0.05 (0.04)	1.05	0.192
Marital status: married	− 0.27 (0.31)	0.76	0.380	− 0.37 (0.32)	0.69	0.258	− 0.18 (0.31)	0.84	0.573	− 0.30 (0.32)	0.74	0.340	− 0.27 (0.32)	0.76	0.383
Job: occupied	− 0.25 (0.35)	0.78	0.468	− 0.27 (0.37)	0.76	0.465	− 0.20 (0.35)	0.82	0.577	− 0.21 (0.36)	0.81	0.567	− 0.23 (0.36)	0.80	0.527
Smoker	− 0.07 (0.32)	0.94	0.838	0.04 (0.34)	1.04	0.900	− 0.08 (0.32)	0.92	0.801	− 0.11 (0.33)	0.90	0.747	− 0.12 (0.33)	0.89	0.712
Alcohol	− 1.12 (0.31)	0.33	< 0.001**	− 1.00 (0.33)	0.37	0.002**	− 1.17 (0.31)	0.31	< 0.001**	− 1.20 (0.32)	0.30	< 0.001**	− 0.98 (0.32)	0.38	0.002**
BMI	0.01 (0.03)	1.01	0.821	0.01 (0.04)	1.01	0.850	0.01 (0.03)	1.01	0.826	0.00 (0.03)	1.00	0.974	0.00 (0.03)	1.00	0.882
Burning				1.02 (0.28)	2.77	< 0.001**									
Itching				0.80 (0.44)	2.23	0.069									
Tingling				0.68 (0.51)	1.97	0.183									
Retromolar Trigone							− 0.38 (0.57)	0.68	0.500						
NRS										0.28 (0.05)	1.33	< 0.001**			
T-PRI													0.23 (0.05)	1.26	< 0.001**
HAM-D															
HAM-A															
PSQI															
ESS															
*R*^2^ (%)	5.5		0.015*	11.6		< 0.001**	7.3		0.004**	15.1		< 0.001**	15.2		< 0.001**
*R*^2^ change (%)				6.1		< 0.001**	1.8		0.044*	9.6		< 0.001**	9.7		< 0.001**

Predictors	Model 6			Model 7			Model 8			Model 9			Model 10		
	Beta (SE)	OR	*p* value	Beta (SE)	OR	*p* value	Beta (SE)	OR	*p* value	Beta (SE)	OR	*p* value	Beta (SE)	OR	*p* value
Age	− 0.01 (0.02)	0.99	0.704	0.00 (0.02)	1.00	0.795	− 0.01 (0.02)	0.99	0.769	− 0.01 (0.02)	0.99	0.718	0.01 (0.02)	1.01	0.582
Gender: female	0.16 (0.30)	1.18	0.586	0.02 (0.32)	1.02	0.940	0.00 (0.32)	1.00	1.000	0.25 (0.30)	1.28	0.406	− 0.27 (0.36)	0.77	0.459
Years of education	0.05 (0.04)	1.05	0.210	0.05 (0.04)	1.05	0.198	0.05 (0.04)	1.05	0.259	0.05 (0.04)	1.05	0.205	0.04 (0.04)	1.04	0.363
Marital status: married	− 0.20 (0.32)	0.82	0.534	− 0.38 (0.33)	0.68	0.251	− 0.31 (0.33)	0.74	0.355	− 0.23 (0.31)	0.79	0.463	0.01 (0.37)	1.01	0.986
Job: occupied	− 0.20 (0.36)	0.82	0.582	− 0.37 (0.38)	0.69	0.325	− 0.40 (0.38)	0.67	0.287	− 0.18 (0.35)	0.83	0.611	0.01 (0.42)	1.01	0.982
Smoker	− 0.09 (0.33)	0.91	0.776	− 0.02 (0.34)	0.98	0.961	0.01 (0.34)	1.01	0.985	− 0.03 (0.33)	0.97	0.921	0.07 (0.38)	1.07	0.859
Alcohol	− 1.02 (0.32)	0.36	0.001**	− 1.00 (0.33)	0.37	0.002**	− 1.03 (0.33)	0.36	0.002**	− 1.07 (0.32)	0.34	0.001**	− 0.92 (0.36)	0.40	0.011*
BMI	0.00 (0.03)	1.00	0.896	0.00 (0.03)	1.00	0.936	0.01 (0.03)	1.01	0.883	0.00 (0.03)	1.00	0.987	− 0.02 (0.04)	0.98	0.641
Burning													0.15 (0.35)	1.16	0.672
Itching													0.23 (0.50)	1.26	0.643
Tingling													1.12 (0.58)	3.08	0.053
Retromolar Trigone													0.06 (0.73)	1.06	0.934
NRS													0.18 (0.08)	1.20	0.019*
T-PRI													0.12 (0.07)	1.12	0.070
HAM-D	0.06 (0.02)	1.07	0.002**										0.05 (0.04)	1.05	0.145
HAM-A				0.06 (0.02)	1.06	0.005**							-0.01 (0.03)	0.99	0.691
PSQI							0.15 (0.04)	1.16	< 0.001**				0.06 (0.05)	1.06	0.289
ESS										0.11 (0.04)	1.11	0.003**	0.11 (0.05)	1.12	0.012*
*R*^2^ (%)	8.6		< 0.001**	7.9		< 0.001**	9.4		< 0.001**	8.2		< 0.001**	25.2		< 0.001**
*R*^2^ change (%)	3.1		0.001**	2.4		0.004**	3.9		< 0.001**	2.7		0.002**	19.7		< 0.001**

SE are the standard errors of the beta estimates. The *p* values were obtained from the hypothesis test on the regression coefficients. *Moderately significant .01 < *p* value ≤ .05. **Strongly significant *p* value ≤ .01

*BMI* body mass index, *ESS* Epworth sleepiness scale, *HAM-A* Hamilton rating scale for anxiety, *HAM-D* Hamilton rating scale for depression, *K-OLP* keratotic oral lichen planus, NRS numeric pain intensity scale, *PSQI* Pittsburgh quality index, *T-PRI* total pain rating index.,

**Table 6:** Multivariate logistic regression analysis predicting geographic area (target area Central-south = 1) in 270 nK-OLP patients

Predictors	Model 1			Model 2			Model 3			Model 4			Model 5		
	Beta (SE)	OR	*p* value	Beta (SE)	OR	*p* value	Beta (SE)	OR	*p* value	Beta (SE)	OR	*P* value	Beta (SE)	OR	*p* value
Age	− 0.01 (0.02)	0.99	0.731	0.00 (0.02)	1.00	0.822	− 0.01 (0.02)	0.99	0.590	0.00 (0.02)	1.00	0.842	0.00 (0.02)	1.00	0.818
Gender: Female	− 0.65 (0.28)	0.52	0.018*	− 0.60 (0.29)	0.55	0.041*	− 0.74 (0.29)	0.48	0.011*	− 0.82 (0.29)	0.44	0.005**	− 0.67 (0.29)	0.51	0.020*
Years of education	0.05 (0.03)	1.06	0.121	0.07 (0.04)	1.08	0.049*	0.06 (0.04)	1.06	0.101	0.07 (0.04)	1.07	0.066	0.08 (0.04)	1.08	0.029*
Marital status: married	− 0.27 (0.30)	0.76	0.365	− 0.10 (0.32)	0.91	0.757	− 0.14 (0.31)	0.87	0.652	− 0.32 (0.31)	0.73	0.314	− 0.37 (0.31)	0.69	0.236
Job: occupied	− 0.41 (0.41)	0.66	0.314	− 0.67 (0.43)	0.51	0.122	− 0.43 (0.42)	0.65	0.307	− 0.26 (0.42)	0.77	0.537	− 0.28 (0.42)	0.76	0.509
Smoker	0.30 (0.36)	1.35	0.400	0.27 (0.37)	1.31	0.470	0.27 (0.37)	1.31	0.464	0.13 (0.37)	1.14	0.724	0.15 (0.37)	1.16	0.693
Alcohol	− 1.02 (0.31)	0.36	0.001**	− 1.09 (0.32)	0.34	0.001**	− 0.98 (0.32)	0.38	0.002**	− 1.02 (0.32)	0.36	0.001**	− 0.87 (0.32)	0.42	0.006**
BMI	0.02 (0.03)	1.02	0.509	0.01 (0.03)	1.01	0.759	0.03 (0.03)	1.03	0.455	0.03 (0.03)	1.03	0.374	0.02 (0.03)	1.02	0.647
Globus pharyngeus				0.93 (0.39)	2.52	0.018*									
Itching				0.99 (0.46)	2.69	0.032*									
Intraoral foreign body sensation				1.03 (0.47)	2.80	0.028*									
Floor of the mouth							0.28 (0.47)	1.32	0.554						
Retromolar trigone							1.02 (0.48)	2.78	0.032*						
NRS										0.10 (0.04)	1.11	0.019			
T-PRI													0.02 (0.02)	1.02	0.410
HAM-D															
HAM-A															
PSQI															
ESS															
*R*^2^ (%)	5.7		0.009**	11.3		< 0.001**	9.0		< 0.001**	7.6		0.002**	5.9		0.013*
*R*^2^ change (%)				5.6		< 0.001**	3.3		0.006**	1.9		0.034*	0.2		0.413

Predictors	Model 6			Model 7			Model 8			Model 9			Model 10		
	Beta (SE)	OR	*p* value	Beta (SE)	OR	*p* value	Beta (SE)	OR	*p* value	Beta (SE)	OR	*P* value	Beta (SE)	OR	*p* value
Age	0.00 (0.02)	1.00	0.989	0.00 (0.02)	1.00	0.806	− 0.01 (0.02)	0.99	0.736	− 0.01 (0.02)	0.99	0.612	0.00 (0.02)	1.00	0.891
Gender: female	− 0.60 (0.28)	0.55	0.034*	− 0.73 (0.28)	0.48	0.011	− 0.67 (0.28)	0.51	0.016*	− 0.61 (0.28)	0.54	0.030*	− 0.76 (0.33)	0.47	0.020*
Years of education	0.08 (0.04)	1.08	0.036*	0.06 (0.04)	1.06	0.080	0.06 (0.03)	1.06	0.113	0.06 (0.04)	1.07	0.071	0.11 (0.04)	1.11	0.010*
Marital status: married	− 0.33 (0.31)	0.72	0.287	− 0.28 (0.31)	0.75	0.364	− 0.28 (0.30)	0.75	0.348	− 0.36 (0.31)	0.70	0.246	− 0.08 (0.35)	0.93	0.823
Job: occupied	− 0.31 (0.42)	0.73	0.457	− 0.33 (0.42)	0.72	0.422	− 0.40 (0.41)	0.67	0.331	− 0.52 (0.42)	0.60	0.218	− 0.54 (0.48)	0.58	0.266
Smoker	0.22 (0.37)	1.24	0.558	0.28 (0.37)	1.32	0.448	0.31 (0.36)	1.37	0.387	0.24 (0.36)	1.27	0.509	0.00 (0.41)	1.00	0.997
Alcohol	− 0.87 (0.32)	0.42	0.006**	− 1.08 (0.32)	0.34	0.001**	− 1.06 (0.32)	0.35	0.001**	− 0.96 (0.31)	0.38	0.002**	− 0.87 (0.35)	0.42	0.014*
BMI	0.04 (0.03)	1.04	0.308	0.02 (0.03)	1.02	0.517	0.02 (0.03)	1.02	0.466	0.02 (0.03)	1.02	0.596	0.02 (0.04)	1.02	0.581
Globus pharyngeus													0.58 (0.45)	1.78	0.200
Itching													0.76 (0.54)	2.14	0.158
Intraoral foreign body sensation													0.83 (0.52)	2.30	0.111
Floor of the mouth													− 0.13 (0.58)	0.88	0.820
Retromolar Trigone													1.26 (0.57)	3.52	0.027*
NRS													0.06 (0.06)	1.07	0.306
T-PRI													− 0.07 (0.03)	0.93	0.029*
HAM-D	0.08 (0.02)	1.08	< 0.001**										0.05 (0.04)	1.05	0.201
HAM-A				0.07 (0.04)	1.07	< 0.001**							0.01 (0.03)	1.01	0.668
PSQI							0.14 (0.04)	1.15	< 0.001**				0.08 (0.05)	1.09	0.076
ESS										0.08 (0.03)	1.08	0.011*	0.02 (0.04)	1.02	0.545
*R*^2^ (%)	10.0		< 0.001**	9.8		< 0.001**	10.2		< 0.001**	7.7		0.001**	18.8		< 0.001**
*R*^2^ change (%)	4.3		< 0.001**	4.1		< 0.001**	4.5		< 0.001**	2.0		0.009**	13.1		< 0.001**

SE are the standard errors of the beta estimates. The *p* values were obtained from the hypothesis test on the regression coefficients. *Moderately significant .01 < *p* value ≤ .05. **Strongly significant *p* value ≤ .01

*BMI* body mass index, *ESS* Epworth sleepiness scale,  *HAM-A* Hamilton rating scale for anxiety, *HAM-D* Hamilton rating scale for depression, *nK-OL*P non-keratotic oral lichen planus, NRS numeric pain intensity scale,﻿ *PSQI* Pittsburgh quality index, *T-PRI* total pain rating index

The final full model (model 10) in which all of the variables were entered simultaneously could explain 19.7% and 13.1% of the variance for the K-OLP and nK-OLP patients, respectively.

Overall, the multivariate logistic regression highlighted that the NRS and T-PRI showed the greatest increase in the R2 values for the CS-K-OLP patients (DR2 = 9.6%; *p* value < 0.001**; DR2 = 9.7% *p* value < 0.001**; respectively) as well as for the oral symptoms (globus, itching and intraoral foreign body sensation) and PSQI (DR2 = 5.6%; *p* value < 0.001**; DR2 = 4.5% *p* value < 0.001** respectively).

## Discussion

This multicenter study provides an evaluation of the socio-demographic and clinical characteristics, psychological profile and symptoms in a large cohort of 540 Italian patients affected by OLP, analyzing, for the first time, the similarities and differences between patients with the K-OLP and nK-OLP subtypes living in the geographical areas of the North and Central-South of Italy.

The socio-demographic characteristics recorded in our study are in line with the majority of large series studies published in literature. Indeed, the prevalence of OLP in female patients is more than twice that of men and the mean age of the patients was around 64 years [8, 9, 21]. Surprisingly, a higher level of education in the CS-nK-OLP patients was found while no differences in family situation, employment, BMI and smoking were reported between the patients from the North and Central-South of Italy. 121(44%) N-OLP and 79 (29%) CS-OLP patients were habitual alcohol consumers with a statistically significant difference in the K-OLP patients; indeed, a higher prevalence in alcohol consumption was found in the N-K-OLP patients (64; 47.4%) compared with the CS-K-OLP patients (37; 27.4%).

The prevalence of systemic diseases is higher in the patients with nK-OLP (224; 83%) compared with the K-OLP patients (151; 56%) but no difference was found between the two different geographical areas. Hypertension was the most frequent comorbidity in both the K-OLP and nK-OLP patients (K-OLP: 41%; nK-OLP: 47%) without any difference between the North and Central-South of Italy, a prevalence higher than that found in previous research studies [8, 22]. In addition, despite the fact that in previous studies the prevalence of hepatitis C arrived at 32% in Italian patients with OLP [23, 24], in this study the hepatitis C infection was found in 2.4% (13) of patients with OLP, without any differences in the prevalence between the North and Central-South of Italy.

A statistically significant difference was found in the prevalence of gastro-esophageal reflux disease in the nK-OLP patients of the different geographical areas; indeed, the N-nK-OLP (26.7%) patients reported a higher prevalence compared with the CS-nK-OLP patients (15.6%).

Although no difference was found in the prevalence of systemic comorbidities in the OLP patients living in the different geographical areas, a statistically significant difference in the assumption of systemic drugs in the nK-OLP patients was detected; indeed, the medications intake in the N-nK-OLP patients (78%) was higher compared with the CS-nK-OLP patients (61.5%), suggesting that there may be a greater treatment adherence in the former group of patients.

The most frequently affected locations in this study coincide with those described by most investigators [25–27]. Indeed, the most common oral sites involved by OLP lesions were the buccal mucosa, with a prevalence of 43%, followed by the gingiva (39%), and tongue (37%). A statistically significant difference in the prevalence of lesions of the retromolar trigone was found between the two geographical areas; indeed, the higher prevalence of lesions in this oral site was detected in the N-K-OLP and in CS-nK-OLP patients.

In line with previous studies [4, 28], in this research OLP patients reported a higher prevalence of oral pain, mostly referred to as a burning sensation, with a higher prevalence in the nk-OLP (67%) compared with the K-OLP (49%) patients. Nevertheless, despite the fact that the CS-OLP patients (64%) were more symptomatic compared with the N-OLP patients (52%), because 63% of the CS-K-OLP versus 35.6% of the N-K-OLP patients reported pain/ burning symptoms (*p* value < 0.001**), the differences in the perception of pain/burning between the two different geographical areas were highlighted only in the K-OLP patients (*p* value < 0.001**). In addition, the CS-K-OLP patients reported a higher percentage of itching (16.3%) and tingling sensation (14.1%) compared with the N-K-OLP patients (7.5% and 5.2% respectively) and the CS-nK-OLP patients reported a higher percentage of globus pharingeus (21.5%), itching (17%) and intraoral foreign body sensation (16.3%) compared with the N-nK-OLP patients (11.1%, 6.7% and 6.7% respectively).

Recent studies have suggested that a complex symptomatology in OLP seems to be due to a peripheral and/or central neuropathy, particularly in patients with many sites involved by OLP lesions [4, 29]. Consequently, this could be related to the extension rather than the severity of the disease [4]. Therefore, it is possible to consider that the CS-OLP subgroups of patients, mainly CS-nK-OLP patients, were more symptomatic on account of the greater extension of disease as they presented a statistically significant higher number of sites involved by OLP lesions (*p* < 0.007**).

Moreover, specific symptoms, such as itching, tingling and intraoral foreign body sensation, are considered part of a peripheral somatosensorial neuropathy [30]. Instead, the presence of globus pharingeus could be explained in a different way. Indeed, this has been reported as a symptom associated with the esophageal involvement of OLP, frequently undetected, because endoscopy is not routinely performed in OLP patients; in addition, the same diagnosis of gastro-esophageal reflux is not always supported by instrumental examinations and may be confused with esophageal lichen planus [31].

Recently, Arduino et al. [32] have reported that patients with fewer oral sites involved by OLP lesions presented across the years a higher risk of cancer development because such patients are less likely to undergo routine oral consultations. Therefore, although the painful symptomatology is associated with an impaired quality of life, the presence of oral symptoms could be protective in that pain is the most important motivation for OLP patients to seek help and treatment from a clinician [33].

This study is a secondary analysis of our previous study in which a prevalence of anxiety, 51%, depression, 48%, and sleep disturbance, 50.5%, were found in OLP patients compared with healthy controls. The prevalence of anxiety identified in this study was similar to that reported in a recent metanalysis of De Porras-Carrique (54.7%) while the prevalence of depression then found was lower (31.19%) [5]. The high prevalence of depression in our sample may be explained by virtue of an analysis of Italian data in a recent epidemiological study of the European Observatory on Health System and Policies (2019), in which a higher proportion of the Italian population aged over 65 presented symptoms of depression (41%) compared to the European population (29%) [34].

The difference in the prevalence of mood disorders and pain perception based on geographical provenance has enhanced our previous analysis with unexpected results. Indeed, we found that both the K-OLP and nK-OLP patients living in the Central-South area showed a significantly higher level and greater prevalence of anxiety (169; 62.5%), depression (156; 57%) and sleep disturbance (164; 60.7%) compared with Northern patients (104, 38.5%; 104, 38.5%; 103, 38.1%, respectively).

The logistic regression analysis confirmed that pain was the most important predictor in the CS-K-OLP patients while symptoms such as globus itching, intraoral foreign body sensation and sleep disturbance were the most important predictors in the CS-nK-OLP group.

The mood impairment in the CS patients may be explained in terms of other environmental factors closely linked to the geographical area. From an analysis of a study of geographical epidemiology it is possible to find that pain perception and mental health are frequently related to socio-demographic, economic, social and cultural aspects which may be different between various countries or in different geographical areas of the same country [35]. Indeed, where one lives may be a determinant of a person’s well-being and such well-being may vary from region to region because lifestyle stressors may be different [35].

Although the health of the Italian population is generally good and life expectancy is the second highest in the EU after Spain, a structural inequality separates the North from the Central-South of Italy, especially in terms of socio-economic status (income level, education, employment status and rate of criminality), wealth and infrastructure [34]. This gap affects life expectancy between those living in central-southern and northern regions and can reach up to three years in favor of the latter [34]. Notably, inequalities in health service delivery, in term of access to primary care across regions of the country, is well known and affects the ability of poorer performing regions to provide access to high-quality health care services [36, 37]. Indeed, the population of the northern regions is generally more satisfied than in the past with the hospital care received; in contrast, in the central-southern regions, the level of satisfaction expressed by patients decreased from 1999 to 2017 in almost all regions [37]. Consequently, the percentage of patients treated in a different region from their home region increased from 7% in 2001 to about 8.5% in 2016, with the proportion of patients in the south choosing to be treated in another region being almost twice as high as that in the north [38]. Therefore, taking into account the fact that access to primary care is a strong predictor of the well-being of a patient, it is probable that a poorer well-being, related to a difficulty in gaining access to a routine consultation, may affect symptoms and mood, particularly in patients with OLP living with the fear of cancer development who should therefore be examined at least once a year [39].

Moreover, the differences in the quality of life perceived by individuals across the country may affect their skills in facing life-threatening diseases. This suggestion is supported by the 2019 edition of the annual quality of life survey in which, generally, northern Italian cities are placed in the highest positions in the ranking while especially southern cities feature in the lowest [40].

Overall, these considerations and the findings from our research may explain why CS-OLP patients, especially those with the keratotic phenotype, may present higher levels of anxiety and depression, which in turn may amplify pain perception [41, 42]. Indeed, immunological processes and the central nervous system are reciprocally and significantly modulated through neurotransmitters, hormones and cytokines, in a complex and bidirectional relationship [43].

The findings from this research are exploratory in nature and should be considered in the light of certain limitations. First, due to the cross-sectional design of the study, it is not possible to establish any cause-effect relationship between pain or psychological profile and geographical provenance in Italy. Secondly, for the same reason, as OLP by definition is a chronic disease potentially characterized by periods of well-being and others of unpredictable reactivations of the symptomatology, there could be the potential bias caused by the possible conversion of K-OLP into nK-OLP and vice versa. Thirdly, other socio-demographic factors which may influence the well-being and the overall quality of life of a subject, such as type of job, income and physical activity, were not explored in this research. Finally, as lifestyle and socio-economic conditions differ considerably from country to country, our results, in terms of clinical characteristics, pain perception and psychological profile, may not be applicable to other populations.

## Conclusions

The results of the present research have shown that pain and mood disorders are predominant in patients with OLP in the Central-South regions of Italy. OLP patients living in the Central-South are affected by a higher prevalence of pain, anxiety, depression and sleep disturbance, compared with patients living in the Northern area. The differences in oral symptoms and psychological profile between patients may be multifactorial. Clinicians should consider the effects of individual and environmental factors when assessing a patient with OLP, taking into account the disparities in health care and in quality of life of patients living in different geographical areas. An appropriate stress management program administered by a stress management professional to identify better ways to enable patients to cope with stress should be considered in OLP patients, especially those patients with higher stressors such as patients living in the Central-South of Italy. This should assist such patients to improve their quality of life and prevent the relapse of disease. Finally, future research should be directed toward the evaluation of socio-demographic and psychological factors in different populations in order to provide greater knowledge about and a deeper insight into the potential relationship between OLP and mental health.

## Data Availability

The datasets generated and/or analysed during the current study are not publicly available due to privacy reasons, but are available from the corresponding author on reasonable request.
